# Effects of Corporate Social Responsibility and Governance on Its Credit Ratings

**DOI:** 10.1155/2014/305452

**Published:** 2014-10-27

**Authors:** Dong-young Kim, JeongYeon Kim

**Affiliations:** ^1^Gangdong College, Janghowon-eup, Icheon-si, Gyeonggi-do 467-900, Republic of Korea; ^2^Sangmyung University, 20 Hongjimun 2-gil, Jongno-gu, Seoul 110-743, Republic of Korea

## Abstract

This study reviews the impact of corporate social responsibility (CSR) and corporate governance on its credit rating. The result of regression analysis to credit ratings with relevant primary independent variables shows that both factors have significant effects on it. As we have predicted, the signs of both regression coefficients have a positive sign (+) proving that corporates with excellent CSR and governance index (CGI) scores have higher credit ratings and vice versa. The results show nonfinancial information also may have effects on corporate credit rating. The investment on personal data protection could be an example of CSR/CGI activities which have positive effects on corporate credit ratings.

## 1. Introduction

Credit rating represents the credit rating agency's evaluation of qualitative and quantitative information on the credit worthiness of a company or government based on their ability to pay back the debt and the likelihood of default. Credit rating agencies disclose their evaluation results for investors to reflect them in their decision-making. Considering that it is not easy to evaluate all credit risks of corporate bond issuers, the evaluation results from credit rating agencies provide an investment guideline to market participants.

Even though agency may reflect additional nonpublic information in the evaluation, its credit rating is primarily based on the announced financial reports. It means that the reliability of accounting information is a critical factor to protect investors from credit risks. There were many efforts to improve the reliability of accounting information. After the experience of accounting frauds such as Enron case, US enacted the Sarbanes-Oxley Act to ensure the financial soundness of listed companies. Korean stock market also has experienced several accounting fraud cases, which were considered as the primary cause of “Korea Discount” phenomenon during last few decades and brought in many policies to verify corporates' accounting information including external audit requirements.

Although company's financial record is a primary factor to evaluate its credit rating, we can think of additional nonfinancial factors that have effects on company's credit rating. One of them is corporate social responsibility (CSR), a form of corporate self-regulation integrated into a business model. It embraces all business management activities beyond legal compliance based on ethics and morality of managers and stakeholders. In addition to the internal benefits of CSR activities such as better corporate image or sustainable management, CSR activities alleviate the risk of information asymmetry between investors by announcing reliable information. CSR activities also may reduce the company's ethical responsibility on its bankruptcy risks by providing investors with proper information on it.

Another example of nonfinancial information affecting the credit ratings is corporate governance. It refers to the mechanisms, processes, and relations by which corporations are controlled and directed. Corporate governance index (CGI) is the evaluation result of the company's internal environment and control system. Proper corporate governance enables reducing corporate's bankruptcy risks and providing useful, timely, and reliable information to the public.

In developed countries, CSR and corporate governance information are assumed as an important part of financial information because they enable investor to estimate the sternness of company's internal control system and the accuracy of company's bankruptcy risk.

The purpose of this study is to verify if the evaluation on CSR and corporate governance is reflected into agencies' credit ratings in Korean money market and to review the correlations between them. This study can be differentiated from previous researches by the fact that we analyze effects on credit rating system of corporate aspects including both financial and nonfinancial information. In addition, the results verify that nonfinancial information such as CSR or corporate governance can be a proxy for credit rating based on the financial efficiency.

## 2. Theoretical Background 

### 2.1. Credit Rating System

Credit ratings are usually attached to debt security such as a bond. Ratings are assigned by credit rating agencies using letter designations such as A, B, and C. Higher grades are intended to represent a lower probability of default. Related researches tried to identify corporate's components or elements which can affect its credit rating.

Standard & Poor's [1] published a report on how to evaluate corporate governance and governance practices. The report suggested 4 important factors on evaluating corporate governance: ownership structure, shareholder rights, financial transparency, and board structure. Individual characteristics of the factors enable effective decision-making by giving a control to managers' behavior and reducing the information asymmetry. The report also insisted identified factors can be used as a proxy of company's credit rating.

Moody's [2] reported that financial credit ratings and corporate governance are related to financial reporting quality, liquidity, and risk management. The expertise, vitality, and independency of board and audit committee can minimize the errors on the cash flow predictability and asset's measurement. Activities of board and audit committee are useful for the control and surveillance on management.

Bhojraj and Sengupta [3] analyzed the relationship between corporate governance and credit ratings. The results showed that companies with higher ratio of outside directors and institutional investment usually have higher credit ratings.

Dillenburg et al. [4] reported that credit rating agencies consider not only financial performance but also environmental, social, and ethical characteristics of corporate in their evaluation. Anderson et al. [5] analyzed the relationship between the agency costs and corporate governance. The independence of the board and audit committee results in higher credit rating and lower interest rates.

### 2.2. Corporate Social Responsibility

CSR-related studies have been done in the various fields of sociology, ethics, or economics. In the field of Business Administration, it was approached as a topic of human resource management and marketing instead of accounting field.

Carroll [6] had focused on ethical approaches on the relationship between business and society. He reviewed the corporates' social responsibility in terms of resolving social problems such as environmental improvement, community-support activities, and sustainable development.

Wood and Jones [7] reviewed the business activities with respect to the corporate responsibility to consumer, community, and other social issues. Creyer [8] insisted that the corporate stakeholders' behavior could be different according to the given expectations for ethical behavior. Ebner and Baumgatner [9] introduced the concept of sustainable development and reviewed the relationship between CSR activities and company's long-term growth. Kotler and Nancy [10] classified CSR activities into social contribution activities, consumer protection activities, and environmental protection activities. They reviewed the CSR effects based on each section.

International Standards Organization announced ISO 26000 as an international standard of corporate's social responsibility in November 1, 2010. It is the final output of efforts to integrate diverse aspects of organizations including ethics, management, and environment.

According to the research of Anderson and Cunningham [11], customers' conception on CSR is different according to his social status and demographic characteristics. Becker-Olsen et al. [12] found that customers reaction to CSR or purchase intention depends on the appropriateness between CSR activities and corporate's business area. Also the corporate's motivation of CSR-related activities, profits-oriented or community-support, affected the customers' evaluation. The analysis of Waddock and Graves [13] also supported the relationship between company's social performance and financial performance.

### 2.3. Corporate Governance

Governance structures identify the distribution of rights and responsibilities among different participants. It also includes the rules and procedures for making decisions in corporate affairs. There has been renewed interest in the corporate governance practices since the several stock market collapses during 2001-2002, most of which involved accounting fraud.

Cohen and Hanno [14] reviewed the effects of alignment on the management philosophy and governance control. In their analyzed preaudit planning, the auditor prefers to contract with the corporate having proper management philosophy and excellent governance structure. Asare et al. [15] showed that the audit fees are actually increased for companies with weak corporate governance structure by analyzing empirical data. Weak governance structure increases audit risk and audit fees are increasing according to it. Others [16, 17] reviewed the relationship between corporate governance and audit fees. They interpreted the risks of audit as higher probability of earning management, which can be a result of weak governance [16].

Kim [18] analyzed the impact of corporate governance on the credit rating. He used the evaluation method from S&P on corporate governance. The results showed that the institutional invest ratio, the quality of accruals, the timeliness of earnings, and the independence of board have positive (+) relations with corporate's credit rating.

## 3. Hypothesis 

### 3.1. Hypothesis Development

If a company actively pursues corporate social responsibility, many researchers expect that its business healthiness and fairness also improve. In addition, the advanced social services and excellent employees' satisfaction by contributing to economic development will lead the company to higher firm value in market. Also the mechanism of corporate governance protects the rights of shareholders and reduces the risk of bankruptcy by resolving the concerns of agency costs and asymmetric information.

The reported cases from other countries verify that CSR activities and corporate governance both should be considered to improve corporate's financial outcome. Many investors concern the risk of bankruptcy, which is related to financial reporting and internal control systems [19]. In Korea, investors also worry about issues of agency problems caused by separation of ownership and management. Logically, the CSR activities and corporate governance should play an important role in Korean capital market. Both of them improve overall efficiency of corporate by the alleviation of agency problem and the announcement of reliable financial information.

Many studies provide verification of their relationships. It would be clear that companies with excellent financial performance would have a higher credit rating. However, the relations between credit rating and nonfinancial information such as CSR and corporate governance are depending on market participants. Capital markets in developed countries are sensitive to the nonfinancial information in addition to the financial results.

The purpose of this study is to analyze the effects on credit rating system of nonfinancial information, CSR, and corporate governance, focusing on the Korean capital market. We set the following hypotheses on the effects of them on corporate credit ratings.

**Hypothesis 1 .**

CSR score has a significant positive (+) impact on the corporate's financial credit rating.

**Hypothesis 2 .**

Corporate governance score has a significant positive (+) impact on the corporate's financial credit rating.

### 3.2. Regression Model

To verify the effects of CSR and corporate governance on corporate credit rating, we first identify corporate's financial aspects having an effect on its credit rating evaluation. Previous researches suggested that financial variables having impacts on the credit rating are assets, inventory change, accounts receivable change, return over asset rate change, and debt ratio change. Adding a variable for CSR and corporate governance each, we can get (1) and (2) for regression analysis. Besides, industry dummy and dummy for year are added to identify their side effects:

(1) CFRi,t=β0+β1∗CSRi,t+β2∗ΔASSTi,t+β3∗ΔRECi,t+β4∗ΔINVi,t+β5∗ΔROAi,t+β6∗ΔDEBTi,t+ΣIDi+ΣYR+εi,t.

(2) CFRi,t=β0+β1∗CGIi,t+β2∗ΔASSTi,t+β3∗ΔRECi,t+β4∗ΔINVi,t+β5∗ΔROAi,t+β6∗ΔDEBTi,t+ΣIDi+ΣYR+εi,t.

(Cf)

 CFR_*i*,*t*_: corporate *i*'s financial credit rating at time *t*;
 CSR_*i*,*t*_: corporate social responsibility index of corporate *i* at time *t*;
 CGI_*i*,*t*_: corporate governance index of corporate *i* at time *t*;
 ΔASST_*i*,*t*_: corporate *i*'s changes of total assets, (ASST_*i*,*t*_ − ASST_*i*,*t*−1_)/ASST_*i*,*t*−1_;
 ΔREC_*i*,*t*_: corporate *i*'s changes of account receivables, (REC_*i*,*t*_ − REC_*i*,*t*−1_)/REC_*i*,*t*−1_;
 ΔINV_*i*,*t*_: corporate *i*'s changes of inventory assets, (INV_*i*,*t*_ − INV_*i*,*t*−1_)/INV_*i*,*t*−1_;
 ΔROA_*i*,*t*_: corporate *i*'s changes of return on assets, (ROA_*i*,*t*_ − ROA_*i*,*t*−1_)/ROA_*i*,*t*−1_;
 ΔDEBT_*i*,*t*_: corporate *i*'s changes of debts, (DEBT_*i*,*t*_ − DEBT_*i*,*t*−1_)/DEBT_*i*,*t*−1_;
 ID: dummy variable for company group;
 YR: dummy variable for year;
 
*ε*_*i*,*t*_: error.

As described before, we identified corporate aspects including both financial and nonfinancial information as independent variables affecting its credit rating, which can be a differentiation from other related researches. We can clearly identify the effects of nonfinancial information on corporate's credit ratings compared to that of financial information.

### 3.3. Data

For the data analysis, we use the credit rating scores published by NICE, a credit rating agency in Korea (http://www.nicerating.com/). NICE credit rating is the result of their integrated model of insolvency prediction and financial evaluation. It refines data categories based on the industry and the credibility intervals based on company assets and continuous holding period.

The financial rating is scored out of 100 points based on the financial statements of the company and additional nonfinancial items.  Table 1 shows the metrics for credit rating. In data analysis, we use natural log of the total score to control heteroscedasticity of each item.

As a proxy of CSR evaluation, we used KEJI Index published by Citizen's Coalition for Economic Justice (CCEJ, http://www.ccej.or.kr/) in Korea.  Table 2 shows their metrics for CSR.

As a proxy of corporate governance, we used the evaluated scores on it during 2007–2009 provided by “Corporate Governance Service” (http://www.cgs.or.kr/) established by the Ministry of Strategy and Finance in Korean Government.  Table 3 shows their metrics for corporate governance.

Among the listed companies, we select candidates whose accounting period is January to December. Empirical data set is the annual financial report during 2007–2009 from selected nonfinancial company. Among 612 companies, we could review financial reports for the period, and we selected 440 companies of which CSR and CGI data were available from the data providers. After removing 1% outliers, we had total 436 data for regression analysis.  Table 4 indicates the distribution of data classified with industry and year.

## 4. Results 

The descriptive statistics of the variables are shown in Table 5. The mean and the standard deviation of financial credit rating, CFR, are 68.128 and 11.415 each. CSR's minimum and maximum values are 0.000 and 74.350, while its average is 21.493. CGI's minimum and maximum values are 20 and 100, while its average is 41.589.

Table 6 shows the correlation between the variables using Pearson correlation coefficients. Both CFR/CSR and CFR/CGI have positive (+) correlation at 1% significant level. Besides, CFR/ΔASST also shows positive correlation significant at the 1% level and CFR/ΔDEBT also shows negative (−) correlation significant at the 5% level.

Those results show credit rating values increases according to the company's asset increase or debt decrease. However, the rate changes of inventory, accounts receivable, and return on asset do not have any significant correlation with corporate credit rating.

The results of regression analysis with (1) and (2) to verify the effects of CSR and corporate governance on credit rating are shown in Table 7. Result for (1) has 0.176 for adjusted *R* ^2^ and its *F*-value is significant. The coefficient of CSR has positive sign and is significant at 1% level, which means that company with higher evaluation on CSR has higher credit rating.

Result for (2) shows that adjusted *R* ^2^ is 0.098 and its *F*-value is also significant. The coefficient of CGI has positive sign and is significant at 5% level, which means that company with higher evaluation on CGI has higher credit rating.

## 5. Conclusion 

This study verifies the relationship between credit rating and nonfinancial information such as CSR and corporate governance. CSR activities have effects of reducing agency costs by eliminating the information asymmetry between internal and external stakeholders. Companies can fundamentally improve their social responsibilities with public announcement of proper financial reports and investment risks. Corporate governance is a key element of the internal control system, which is also critical to provide timely and reliable corporate financial information for outside investors to accurately judge the bankruptcy risk of the company. Both of them help to reduce agency costs and minimize the unfavorable side effect of information asymmetry.

Traditionally, corporate's financial information and credit rating are intimately related. The suggested nonfinancial information has an effect on corporate credit rating individually, but integrated verification also considering the financial information is not provided for Korea market yet.

To prove our assumptions of nonfinancial information on the relationship, we provided empirical analysis with data from selected companies. The results of the analysis showed a significant correlation between credit rating and CSR and corporate governance. In the regression analysis between related variables, coefficients had positive (+) sign for both of them. Also the *F*-value of regression results was statistically significant at the 1% level. As we assumed, a company with higher evaluation on CSR and corporate governance has solid credit rating score. The effects of suggested nonfinancial information are positive on corporate credit rating regardless of the effects of financial information.

We interpret the analysis results as follows. Credit rating system in Korea market is mutualized as in other developed countries. The agents' index calculation methods are stabilized and we can predict the reaction of investors in stock markets according to the credit rating disclosure. The coherence between corresponding CSR/CGI variables and credit ratings has been increased during few decades and the results of data analysis between those variables prove their relationships.

The higher the credit rating has a corporate, the better the scores of corporate governance and corporate social responsibility are expected. Additional analysis for the refined metrics of CSR and corporate governance could provide investors with specific insights into the proxy of their market value. For the further research of CSR, we could consider wider nonfinancial area such as data security on personal information.

## Figures and Tables

**Table 1:** Criteria of credit rating.

Items	Variables	Assigned scores
Stability	Ratio of net worth to total capital	10
	Debt/sale	10

Liquidity	Sale/NCR (net capital ratio)	10
	Current ratio	10

Profitability	Ratio of net income to total capital	10
	Financial cost burden	10

Growth	Sales growth	5
	Total assets growth	5

Activity	Total assets turnover ratio	5
	Accounts receivable turnover ratio	5

Size	Total assets	7.5
	Sales	7.5

Nonfinancial	Corporate type	3
	Others	2

Total		100

**Table 2:** Evaluation item for CSR.

Items	Evaluation criteria
Healthiness	Shareholder composition, soundness of spending/investment and capital raise

Fairness	Fairness, transparency, relationship subcontract

Public contribution	Underprivileged protection, social welfare support

Consumer protection	Consumer rights protection, quality, advertising

Environmental protection	Environmental improvements result, violations, and pollution performance

Employee welfare	Industrial accidents, human resource investment, wage benefits, labor relations, equal employment

Economic development	Research and development efforts, financial performance, and economic contribution

**Table 3:** Criteria for corporate governance.

Items	Description
Rights of shareholders	Introduction of the corporate governance charter and code of ethics Introduction of cumulative voting or written voting Introduction of staggered election

Board of directors	Outside directors and attendance ratio Outside directors' objections or suggested modifications Recommendation of outside directors

Disclosure	IR performance Disclosures frequency including voluntary, queries, and corrections Announcement of the board member's attendance and voting

Audit committee	Organization of the audit committee, the configuration, and operating Establishment of an internal crime reporter protection Recommendation of external auditors or consulting

Reward management	Dividend yield, share buyback Three-year average dividend payout ratio, interim dividend

**Table 4:** Data group categorised by industry.

Industry	2007	2008	2009	Total
Food/drug, textile, paper	31	30	34	95
Metal/nonmetal, chemistry	32	31	35	98
Electronics, machinery	43	45	43	131
Service	37	36	39	112
All	**143**	**142**	**151**	**436**

**Table 5:** Statistics of variables.

	Min.	Max.	Average	SD	Count
CFR	30	95	68.128	11.415	436
CSR	0	74.35	21.493	29.965	436
CGI	20	100	41.589	11.809	436
ΔASST	−0.89	0.869	0.006	0.172	436
ΔREC	−0.803	1.96	0.113	0.353	436
ΔINV	−0.945	1.986	−0.393	0.339	436
ΔROA	−6.928	7.502	−0.135	1.47	436
ΔDEBT	−0.467	0.954	0.025	0.184	436

**Table 6:** Pearson correlation between variables.

	CFR	CSR	CGI	ΔASST	ΔREC	ΔINV	ΔROA	ΔDEBT
CFR	1							
CSR	0.060∗∗∗	1						
CGI	0.180∗∗∗	0.195∗∗∗	1					
ΔASST	0.074∗∗∗	−0.017	0.007	1				
ΔREC	−0.003	−0.023	0.069∗∗∗	−0.008	1			
ΔINV	−0.009	−0.015	0.038	0.02	0.053	1		
ΔROA	0.016	0.036∗	0.024	−0.004	0.016	0.012	1	
ΔDEBT	−0.007∗∗	−0.012	−0.006	0.01	0.05	0.024	−0.014	1

^*^ *P* < 0.05, ^**^ *P* < 0.01, and ^***^ *P* < 0.001.

**Table 7:** Results of regression analysis with equations.

	Equation (1)	Equation (2)
CSR	0.294 (6.638∗∗∗)	
CGI		0.093 (2.013∗∗)
ΔASST	−0.013 (−0.228)	0.005 (0.118)
ΔREC	0.106 (2.285∗∗)	0.097 (2.007∗∗)
ΔINV	0.209 (4.594∗∗∗)	0.230 (4.840∗∗∗)
ΔROA	0.145 (3.265∗∗∗)	0.161 (3.465∗∗∗)
ΔDEBT	0.294 (−2.709∗∗∗)	−0.123 (−2.550∗∗)
ID	Included	Included
YR	Included	Included
*F* value	16.149∗∗∗	8.722∗∗∗
Adj *R* ^2^	0.176	0.098

Cf:  *N* = 436; *t*-value in ( ).

^*^ *P* < 0.05, ^**^ *P* < 0.01, and ^***^ *P* < 0.001.
